# Contribution of thrombin-reactive brain pericytes to blood-brain barrier dysfunction in an *in vivo* mouse model of obesity-associated diabetes and an *in vitro* rat model

**DOI:** 10.1371/journal.pone.0177447

**Published:** 2017-05-10

**Authors:** Takashi Machida, Fuyuko Takata, Junichi Matsumoto, Tomoyuki Miyamura, Ryosuke Hirata, Ikuya Kimura, Yasufumi Kataoka, Shinya Dohgu, Atsushi Yamauchi

**Affiliations:** 1 Department of Pharmaceutical Care and Health Sciences, Faculty of Pharmaceutical Sciences, Fukuoka University, Fukuoka, Japan; 2 BBB Laboratory, PharmaCo-Cell Co., Ltd., Nagasaki, Japan; Washington University, UNITED STATES

## Abstract

Diabetic complications are characterized by the dysfunction of pericytes located around microvascular endothelial cells. The blood–brain barrier (BBB) exhibits hyperpermeability with progression of diabetes. Therefore, brain pericytes at the BBB may be involved in diabetic complications of the central nervous system (CNS). We hypothesized that brain pericytes respond to increased brain thrombin levels in diabetes, leading to BBB dysfunction and diabetic CNS complications. Mice were fed a high-fat diet (HFD) for 2 or 8 weeks to induce obesity. Transport of i.v.-administered sodium fluorescein and ^125^I-thrombin across the BBB were measured. We evaluated brain endothelial permeability and expression of tight junction proteins in the presence of thrombin–treated brain pericytes using a BBB model of co-cultured rat brain endothelial cells and pericytes. Mice fed a HFD for 8 weeks showed both increased weight gain and impaired glucose tolerance. In parallel, the brain influx rate of sodium fluorescein was significantly greater than that in mice fed a normal diet. HFD feeding inhibited the decline in brain thrombin levels occurring during 6 weeks of feeding. In the HFD fed mice, plasma thrombin levels were significantly increased, by up to 22%. ^125^I-thrombin was transported across the BBB in normal mice after i.v. injection, with uptake further enhanced by co-injection of unlabeled thrombin. Thrombin-treated brain pericytes increased brain endothelial permeability and caused decreased expression of zona occludens-1 (ZO-1) and occludin and morphological disorganization of ZO-1. Thrombin also increased mRNA expression of interleukin-1β and 6 and tumor necrosis factor-α in brain pericytes. Thrombin can be transported from circulating blood through the BBB, maintaining constant levels in the brain, where it can stimulate pericytes to induce BBB dysfunction. Thus, the brain pericyte–thrombin interaction may play a key role in causing BBB dysfunction in obesity-associated diabetes and represent a therapeutic target for its CNS complications.

## Introduction

The increasing prevalence of type 2 diabetes represents a major global health issue because of the numerous and often serious complications associated with it [1], including nephropathy, retinopathy and neuropathy [2]. Type 2 diabetes is a chronic systemic inflammatory disease; this inflammation is characterized by infiltration of macrophages into adipose tissue, where they produce pro-inflammatory cytokines, including tumor necrosis factor-α (TNF-α), interleukin (IL)-1β and IL-6, which contribute to insulin resistance [3]. An inflammatory process is also involved in the development and progression of diabetic complications [2, 4, 5]. In addition to the peripheral complications mentioned above, complications in the central nervous system (CNS), such as diabetic cognitive impairment, vascular dementia and Alzheimer’s disease (AD), can occur in diabetes [6–11].

The common event underlying these diabetic complications is local microvascular failure [12, 13]. A characteristic feature of microvascular structure is the pericytes located around microvascular endothelial cells; these perivascular cells are known as podocytes in the kidney, retinal pericytes in the retina, and brain pericytes in the brain. Loss of pericytes causes microvascular diabetic complications; diabetic-induced albuminuria and diabetic retinopathy likely occur due to impaired podocyte [14, 15] and retinal pericyte [16, 17] function, respectively. In addition to these peripheral pathologies, loss of brain pericytes has been proposed to occur in diabetes, and indeed a recent study reported loss of pericytes in the brain of diabetic mice [18].

Brain pericytes are a constituent of the blood–brain barrier (BBB), along with brain microvascular endothelial cells (BMECs) and astrocytes. The BBB has a highly specialized structure designed to regulate transmission of substances between the brain and the circulation. Brain pericytes are located adjacent to capillaries and share a common basement membrane with BMECs. To regulate BBB function, brain pericytes communicate directly or indirectly with BMECs through gap junctions or soluble factors [19–21]. Decreased numbers of pericytes in the brain microvasculature is associated with disruption of the BBB in patients with mild cognitive impairment [22]. Dysfunction and hyperpermeability of the BBB is also a feature of diabetes progression [23, 24], while neurological diseases, such as AD, involve disruption of the BBB [25–28]; hence, diabetes is considered to be a risk factor for vascular dementia and AD. Therefore, brain pericyte dysfunction may be important for the development of diabetic complications in the CNS, including BBB and cognitive impairment. However, the direct cause of diabetes-related BBB dysfunction and the importance of brain pericytes in this remain to be established.

Impaired integrity of the BBB is a prerequisite for diabetes-related neurological disorders. Both *in vitro* and *in vivo* studies have shown that hyperglycemia, advanced glycation end-products, matrix metalloproteinases (MMPs), and inflammatory cytokines are involved in diabetes-induced BBB dysfunction [23, 29, 30]. However, diabetes is also a hypercoagulant state, reflected by elevated plasma levels of coagulation factors, including thrombin. Thrombin is an essential component of the coagulation cascade that produces fibrin clots, but in addition to its central role in the coagulation process, thrombin works as an inflammatory mediator through its specific receptors, protease-activated receptors (PARs). Blood thrombin is increased in diabetes [31] and has also been detected in microvessels isolated from the brains of AD patients [32]. Furthermore, several *in vitro* studies have shown that thrombin acts directly on BMECs to increase BBB permeability [33–35]. We have previously shown that brain pericytes are the most thrombin-sensitive cell type of the BBB, releasing MMP-9 after binding of thrombin to PARs [36]. Considering these findings, we hypothesize that brain pericytes could respond adversely to increased brain levels of thrombin during diabetes, leading to BBB dysfunction and diabetic CNS complications.

In the present study, we have determined whether there is an interaction between thrombin and brain pericytes under obesity-associated diabetic conditions. We investigated thrombin generation in the brain of mice with high-fat diet (HFD)-induced obesity and the involvement of brain pericytes in thrombin-induced BBB dysfunction using an *in vitro* model of the BBB. We present evidence that brain pericytes could respond to increased brain thrombin in obesity-associated diabetes to develop BBB dysfunction and consequently diabetic CNS complications.

## Materials and methods

### Mouse model of high-fat diet-induced obesity

All procedures involving animals adhered to the law (No. 105) and notification (No. 6) of the Japanese Government, and were approved by the Laboratory Animal Care and Use Committee of Fukuoka University. Four-week-old male Jcl:ICR mice were purchased from Kyudo Experimental Animal Laboratory (Tosu, Japan). Mice were housed at a controlled temperature (22 ± 2°C) under a 12 h light/dark cycle (lights on from 07:00 to 19:00 h), with free access to water and normal chow diet. After habituation for one week, mice were divided into two groups: a normal diet (ND) group and a HFD group. Obesity was induced by feeding of High-fat Diet-32 (Clea Japan, Tokyo, Japan) for 2 weeks (2W) or 8 weeks (8W), while ND mice were fed diet CE-2 (Clea Japan). Fasting blood insulin, leptin, triglyceride and total cholesterol levels were measured using a mouse insulin ELISA kit (Morinaga Institute of Biological Science, Inc., Yokohama, Japan), mouse leptin ELISA kit (Morinaga), triglyceride E test kit (Wako, Osaka, Japan) and total cholesterol E test kit (Wako), respectively.

### Oral glucose tolerance test (OGTT)

Blood was collected from a tail vein of overnight-fasted mice, then fasting blood glucose (t = 0) was measured by the glucose oxidase method using a Glutest sensor and Glutest Ace (Sanwa Kagaku Kenkyusho, Nagoya, Japan). Mice were then gavaged with 40% glucose solution (2 g/kg) and repeat blood glucose measurements were made at 30, 60, and 120 min after glucose administration.

### Measurement of brain uptake of sodium fluorescein

Brain uptake of sodium fluorescein (Na-F) was measured as previously described [37], with a minor modification. Mice were anesthetized by i.p. injection of sodium pentobarbital (78 mg/kg). Two hundred microliters of phosphate buffered saline (PBS) containing 6 mg/mL Na-F was injected into the jugular vein. Blood was obtained from the carotid artery 5, 10, 15 and 20 min after the i.v. injection and mice were immediately decapitated. The whole brain was removed and weighed. Brain/serum ratios were calculated by dividing the amount of Na-F in a gram of brain by the amount of Na-F in a microliter of serum. Brain/serum ratios were plotted against exposure times. The exposure time was calculated as the area under the curve in a plot of serum concentration divided by the serum concentration at time *t*. The linear portion of the slope of the resulting graph represents the unidirectional influx rate (K_in_) (μL/g brain-min) and the y-intercept indicates the distribution space in the brain (V_i_) (μL/g brain) at t = 0.

### ELISA for prothrombin/thrombin

Mice were anesthetized by i.p. administration of 40% urethane (Sigma-Aldrich, St. Louis, MO, USA). Blood was obtained from the right ventricle of the heart and following transcardiac brain perfusion with PBS, brains were dissected. Plasma and brain samples were prepared for ELISA using the sample preparation protocol supplied by Abcam (Cambridge, UK). Levels of Prothrombin/Thrombin in brain and plasma were measured with the Prothrombin/Thrombin Total Mouse ELISA kit (ab157527: Abcam) according to the manufacturer's instructions.

### Unidirectional influx rate of ^125^I-thrombin

Fifteen μg of thrombin or BSA (Sigma), 3.7 MBq of ^125^I-Na (Perkin Elmer, Boston, MA) and 30 μg of chloramine-T (Nacalai Tesque, Kyoto, Japan) were incubated together for 60 sec. The radioactively labeled proteins were separated from free iodine on a column of Sephadex G-10 (Sigma). Adult male ICR mice were anesthetized with an intraperitoneal injection of urethane (40%) and 200 μL of physiological buffer (141 mM NaCl, 4 mM KCl, 2.8 mM CaCl_2_, 1.0 mM MgSO_4_, 1.0 mM NaH_2_PO_4_, 10 mM HEPES and 10 mM d-glucose, pH 7.4) containing 1% BSA and 5.5 × 10^5^ cpm of ^125^I-thrombin or ^125^I-albumin was injected into a jugular vein. Brain and carotid artery blood samples were obtained 1, 3, 5, 10, 30, 60, 120 and 240 min after the i.v. injection (n = 3–4 per time point), arterial blood was collected from the carotid artery and the brain was removed and weighed. For transcardiac brain perfusion, a 27-gauge butterfly needle was inserted into the left ventricle of the heart and physiological buffer, containing 1% BSA and ^125^I-thrombin or ^125^I-albumin (200,000 cpm/mL each), with or without 50 μg/mL unlabeled thrombin, was infused at a rate of 2 mL/min for 0.5–2 min. All samples were mixed with 30% trichloroacetic acid (TCA; Wako), homogenized and then centrifuged at 5,400 × g for 15 min at 4°C. The radioactivity in the TCA precipitate derived from brain and 50 μL of serum was determined using a gamma counter. The K_in_ and V_i_ were determined by multiple time regression analysis as described in “Measurement of brain uptake of sodium fluorescein”.

### Preparation of an in vitro model of the blood–brain barrier

Primary cultures of rat brain microvascular endothelial cells (RBECs) and brain pericytes were prepared from 3-week-old Wistar rats, as previously described [38]. Three-week-old Wistar rats were purchased from Kyudo Experimental Animal Laboratory (Tosu, Japan). Rats were housed at a controlled temperature (22 ± 2°C) under a 12 h light/dark cycle (lights on from 07:00 to 19:00 h), with free access to water and normal diet. For primary cell isolation, the rats were deeply anesthetized with isoflurane (Wako) and decapitated. Brain pericytes (4 × 10^4^ cells/well) were seeded in 24-well culture plates (Costar, Corning, NY, USA). After 2–3 days, RBECs (5 × 10^4^ cells/well) were seeded on fibronectin-collagen IV (0.1 mg/mL)-coated polyester membranes (0.33 cm^2^, 0.4 μm pore size) of Transwell^®^-Clear inserts (Costar) placed in each well of a 24-well culture plate containing layers of brain pericytes (RBEC/pericyte co-cultures). This co-culture system allows cells to communicate with each other through soluble factors. A monolayer system was also established with RBECs alone (RBEC monolayers). Cells were cultured in RBEC medium (Dulbecco’s modified Eagle medium/F12 (Wako)) supplemented with 10% bovine plasma-derived serum (Animal Technologies, Tyler, TX), basic fibroblast growth factor (1.5 ng/mL; Roche), heparin (100 μg/mL; Sigma-Aldrich), insulin (5 μg/mL), transferrin (5 μg/mL), sodium selenite (5 ng/mL; insulin-transferrin-sodium selenite media supplement; Sigma-Aldrich) and gentamicin (50 μg/mL), containing 500 nM hydrocortisone (Sigma-Aldrich), at 37°C in a humidified atmosphere of 5% CO_2_/95% air, until the cells reached confluency. Then, the culture medium was replaced with serum-free RBEC medium. In luminal chambers, the medium contained 500 nM hydrocortisone. At the same time, thrombin (1 or 3 U/mL) was added to abluminal or luminal chambers. After 24 h treatment, the *in vitro* BBB models were assessed for transendothelial Na-F transport, transendothelial electrical resistance (TEER) and subjected to immunostaining or western blot analyses. TEER values were measured with an epithelial-volt-ohm meter and Endohm-6 chamber electrodes (World Precision Instruments, Sarasota, FL, USA). The TEER values of cell-free Transwell inserts were subtracted from the measured TEER values of the RBECs, and are expressed as Ω × cm^2^. Cell viability was assessed using Cell Counting Kit-8 (Dojindo, Kumamoto, Japan), according to the manufacturer's instructions.

### Measurement of paracellular transport of Na-F

To initiate the transport experiments, the medium was removed and physiological buffer containing 100 μg/mL Na-F (Sigma-Aldrich) was loaded into luminal chambers of the insert (0.1 mL). Samples (0.4 mL) were removed from abluminal chambers at 15, 30, 45, and 60 min and immediately replaced with fresh physiological buffer. The concentration of Na-F was determined using a fluorescence multi-well plate reader (excitation wavelength 485 nm; emission wavelength 530 nm; CytoFluor Series 4000; PerSeptive Biosystems, Framingham, MA, USA). The permeability coefficient and clearance were calculated as previously described [39, 40].

### Western blotting and immunofluorescence staining

Western blot and immunofluorescence staining for detection of tight junction (TJ) proteins were performed as previously described [38].

### Quantitative real-time RT-PCR analysis

Real-time quantitative PCR was carried out as previously described [41]. The specific primer-probe sets for rat IL-1β, IL-6, TNF-α and GAPDH were purchased from Takara (Shiga, Japan). All mRNA measurements were normalized to the reference gene, GAPDH. Gene expression levels were calculated using MxPro QPCR Software (Agilent Technologies, Santa Clara, CA, USA).

### Statistical analysis

Results are shown as mean ± S.E.M. Statistical interactions were assessed by two-way of analysis of variance (ANOVA). The statistical significance of differences between groups was assessed by one-way ANOVA for factorial comparisons and by Dunnett's or Tukey-Kramer's test for multiple comparisons, using GraphPad Prism 5.0 (GraphPad, San Diego, CA, USA). Differences were considered significant with P<0.05.

## Results

### Generation of HFD-induced obesity mouse model

As shown in Table 1, mice showed a significant increase in body weight gain after both 2W and 8W of HFD-feeding. Fasting blood glucose level was significantly increased by both 2W and 8W of HFD-feeding compared to ND-fed mice. Blood glucose in mice fed a HFD for 2W was significantly increased at 60 and 120 min after glucose administration and was increased at all time-points after 8W of HFD. The area under the curve for the OGTT (iAUC) was significantly increased in HFD-fed mice after 8W but not 2W of HFD. Fasting blood insulin, leptin and triglyceride levels were significantly higher in mice fed a HFD, compared with ND, at 2W and 8W (Table 2).

**Table 1:** Obesity and glucose tolerance in ND- and HFD-fed mice.

	Body Weight gain (g)	OGTT (mg/dL)				iAUC (mg/dL × min)
		0	30	60	120 (min)	
2W ND	4.7±0.3	33.9±2.6	243.9±24.5	256.2±21.4	122.7±9.7	316.1±29.0
2W HFD	12.2±0.9***	48.4±2.3***	239.2±15.4	334.8±18.7*	189.7±19.5**	380.9±26.8
8W ND	30.5±1.1	48.0±3.2	155.4±11.5	132.1±8.3	78.2±3.9	131.9±11.6
8W HFD	46.2±1.3***	88.4±7.6***	249.3±21.8**	349.9±28.3***	214.9±20.0***	339.8±36.8***

Mice were fed a normal diet (ND) or high-fat diet (HFD) for two (2W) or eight (8W) weeks. Obesity and glucose tolerance are indicated by body weight, fasting blood glucose, oral glucose tolerance test (OGTT), and area under the OGTT curve (iAUC). Fasting blood glucose is indicated by the 0 min sample of the OGTT.

* p<0.05,

** p<0.01,

*** p<0.001 vs. mice fed ND for2W or 8W (n = 9–10).

**Table 2:** Blood chemistry in ND- and HFD-fed mice.

	Fasting insulin (μU/mL)	Leptin (ng/mL)	Triglyceride (mg/dL)	Total cholesterol (mg/dL)
2W ND	12.3±1.2	1.2±0.4	97.3±4.4	102.7±7.1
2W HFD	44.3±8.1**	8.4±1.1***	119.7±5.1*	121.5±8.9
8W ND	7.3±0.5	0.3±0.1	44.0±3.8	84.7±5.7
8W HFD	30.8±4.3**	19.0±2.0***	73.2±7.2*	98.8±6.1

Mice were fed a normal diet (ND) or high-fat diet (HFD) for 2W or 8W.

* p<0.05,

** p<0.01,

*** p<0.001 vs. mice fed ND for 2W or 8W (n = 5–8).

### Brain uptake of Na-F in HFD-fed mice

**Fig 1:**
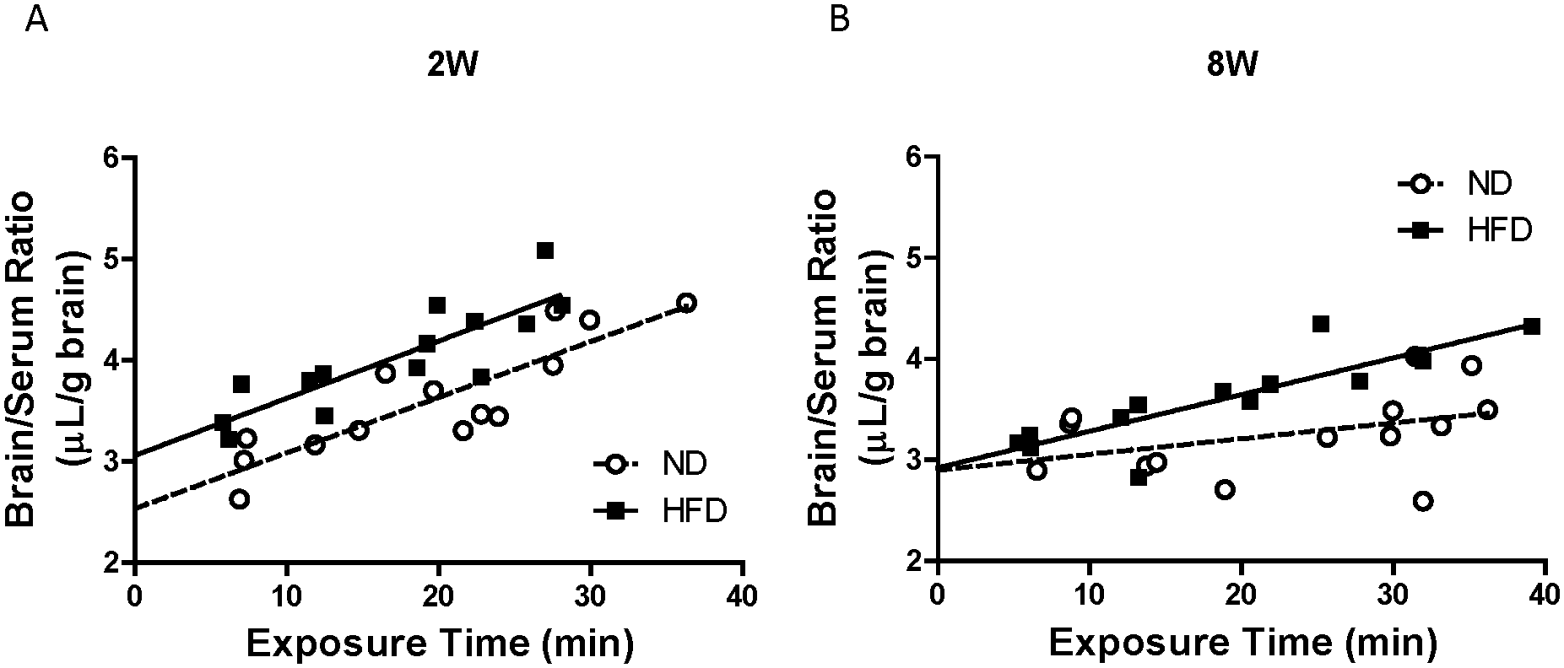
Brain uptake of sodium fluorescein in mice fed ND or HFD for two (2W) or eight (8W) weeks. Mice were injected with 200 μL of physiological buffer containing Na-F (6 mg/mL) i.v. and killed 5, 10, 15 and 20 min later. Each point represents data from one mouse.

The relationship between whole brain/serum ratios and exposure times for Na-F in mice fed a HFD for 2W and 8W is shown in Fig 1. The blood-to-brain unidirectional influx rate (K_in_) values for Na-F were 0.016 ± 0.01 and 0.036 ± 0.006 μL/g brain-min in mice fed ND and HFD, respectively, for 8W. The V_i_ values were 2.9 ± 0.26 and 2.9 ± 0.14 μL/g brain in mice fed a ND and HFD, respectively, for 8W. The K_in_ in mice fed a HFD for 8W was significantly higher than that in ND-fed mice. HFD feeding for 2W had no significant effect on K_in_ for Na-F (ND: 0.055 ± 0.009 μL/g brain-min vs. HFD: 0.056 ± 0.01 μL/g brain-min), but led to an increased V_i_ for Na-F (ND: 2.5 ± 0.19 μL/g brain vs. HFD: 3.1 ± 0.19 μL/g brain).

### Prothrombin/thrombin production in HFD-induced obesity mice

**Fig 2:**
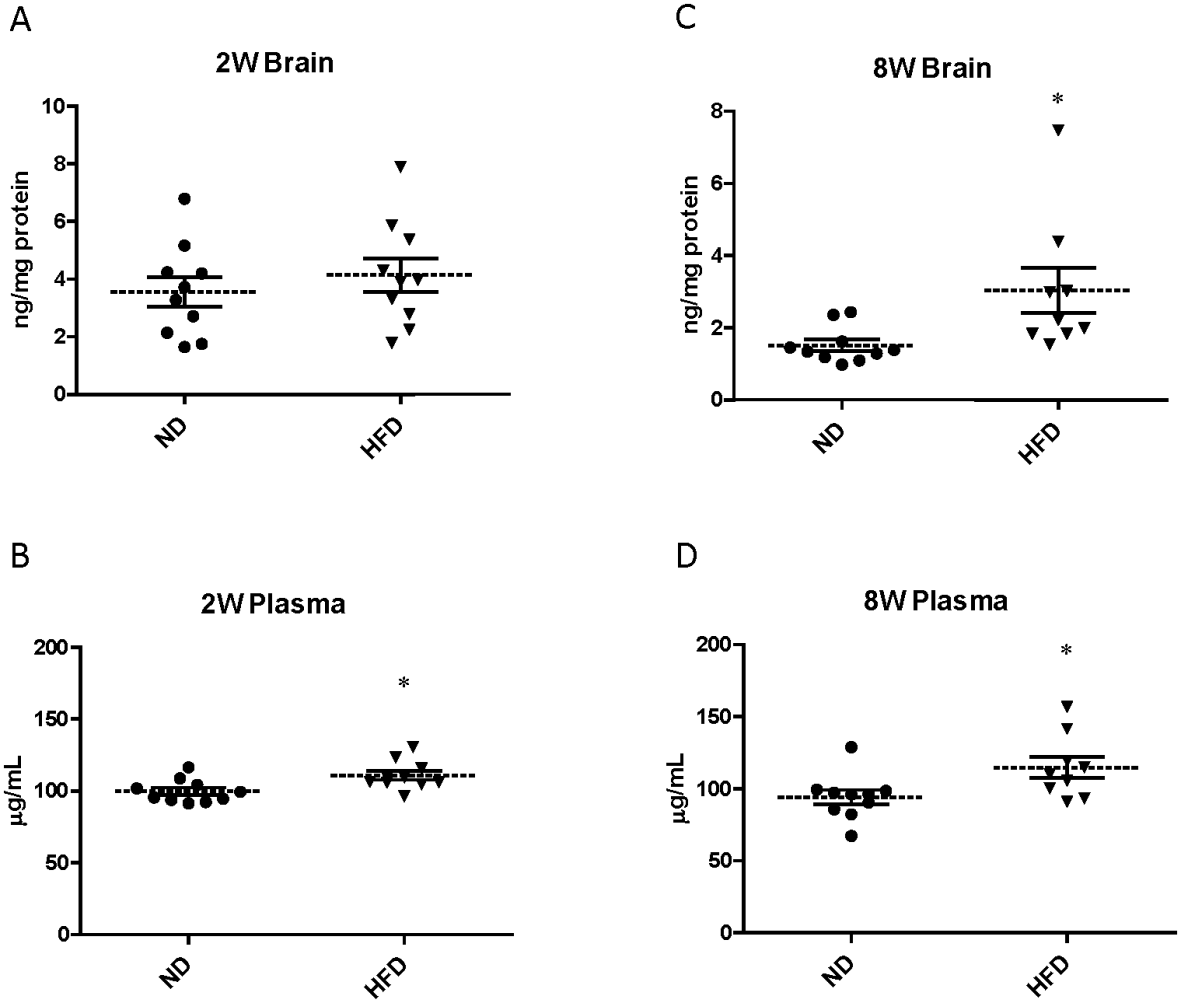
Prothrombin/thrombin levels in brain (A, C) and plasma (B, D) of mice fed ND or HFD for 2W or 8W. Measured using ELISA. Circles represent individual ND mice and triangles HFD mice. Dotted lines represent means and solid lines SEMs (n = 9–10). *p<0.05 vs ND-fed mice.

Protein levels of prothrombin/thrombin in the plasma were significantly increased in mice fed a HFD for 2W by 11% (ND: 99.80 ± 2.56 μg/mL vs. HFD: 110.85 ± 3.14 μg/mL) and for 8W by 22% (ND: 94.23 ± 4.98 μg/mL vs. HFD: 114.82 ± 7.32 μg/mL) (Fig 2B and 2D). Brain prothrombin/thrombin levels in mice fed a ND were decreased from 3.56 ± 0.51 to 1.51 ± 0.16 ng/mg protein over the duration of feeding (Fig 2A and 2C). In contrast, the prothrombin/thrombin content of whole brain in mice fed a HFD for 8W was maintained at a level two-fold higher that in mice fed a ND for 8W (ND: 1.51 ± 0.16 ng/mg protein vs. HFD: 3.03 ± 0.63 ng/mg protein).

### Blood-to-brain transport of ^125^I-thrombin

**Fig 3:**
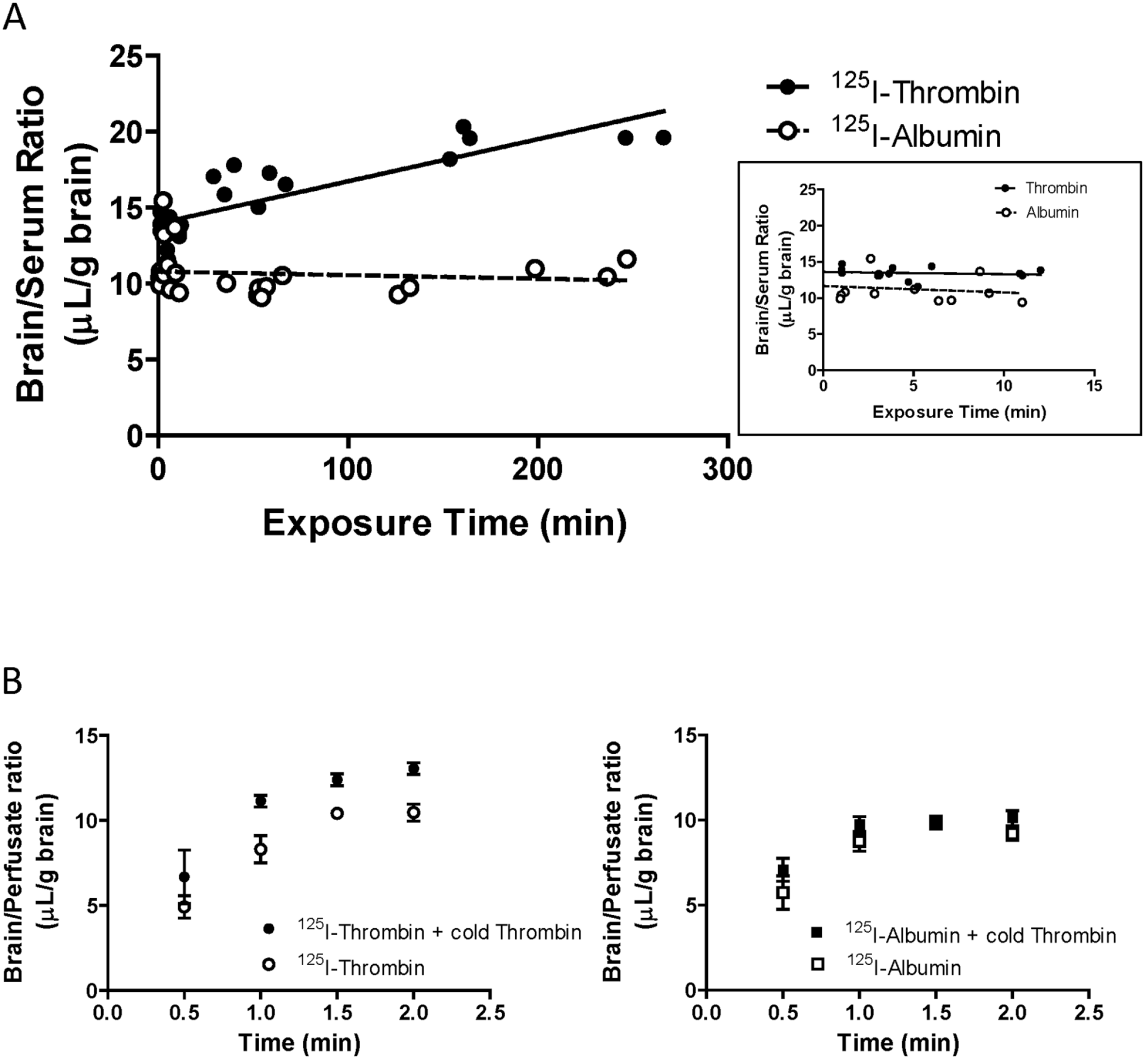
Transport of ^125^I-thrombin across the BBB in normal mice. (A) Multiple time regression analyses of ^125^I-thrombin and ^125^I-albumin versus time after i.v. injection in normal mice. The unidirectional influx rate (K_in_) of ^125^I-thrombin was 0.0278 ± 0.0037 μL/g brain-min. The inset shows brain/serum ratios from 0 to 15 min. Each point represents data from one mouse. (B) Time courses of the brain/perfusate ratios of ^125^I-thrombin (left panel) and ^125^I-albumin (right panel), with or without an excess amount of unlabeled thrombin (50 μg/mL), obtained in normal mice by transcardiac brain perfusion. Data are means ± SEM (n = 3–4 mice per point).

To evaluate whether thrombin can enter the brain from the circulation, we measured the K_in_ of ^125^I-thrombin in normal mice. Fig 3A shows that the brain/serum ratio of ^125^I-thrombin was increased with time after i.v. injection of ^125^I-thrombin. The K_in_ of ^125^I-thrombin was 0.0278 ± 0.0037 μL/g brain-min, which was significantly higher than that of ^125^I-albumin. During transcardiac brain perfusion with an excess amount of unlabeled thrombin (50 μg/mL), brain uptake of ^125^I-thrombin was increased (Fig 3B). Brain uptake of ^125^I-albumin was not altered by excess unlabeled thrombin, showing that the unlabeled thrombin did not affect BBB integrity.

### Effect of thrombin on permeability of RBECs in RBEC monolayers or RBEC/pericyte co-cultures

**Fig 4:**
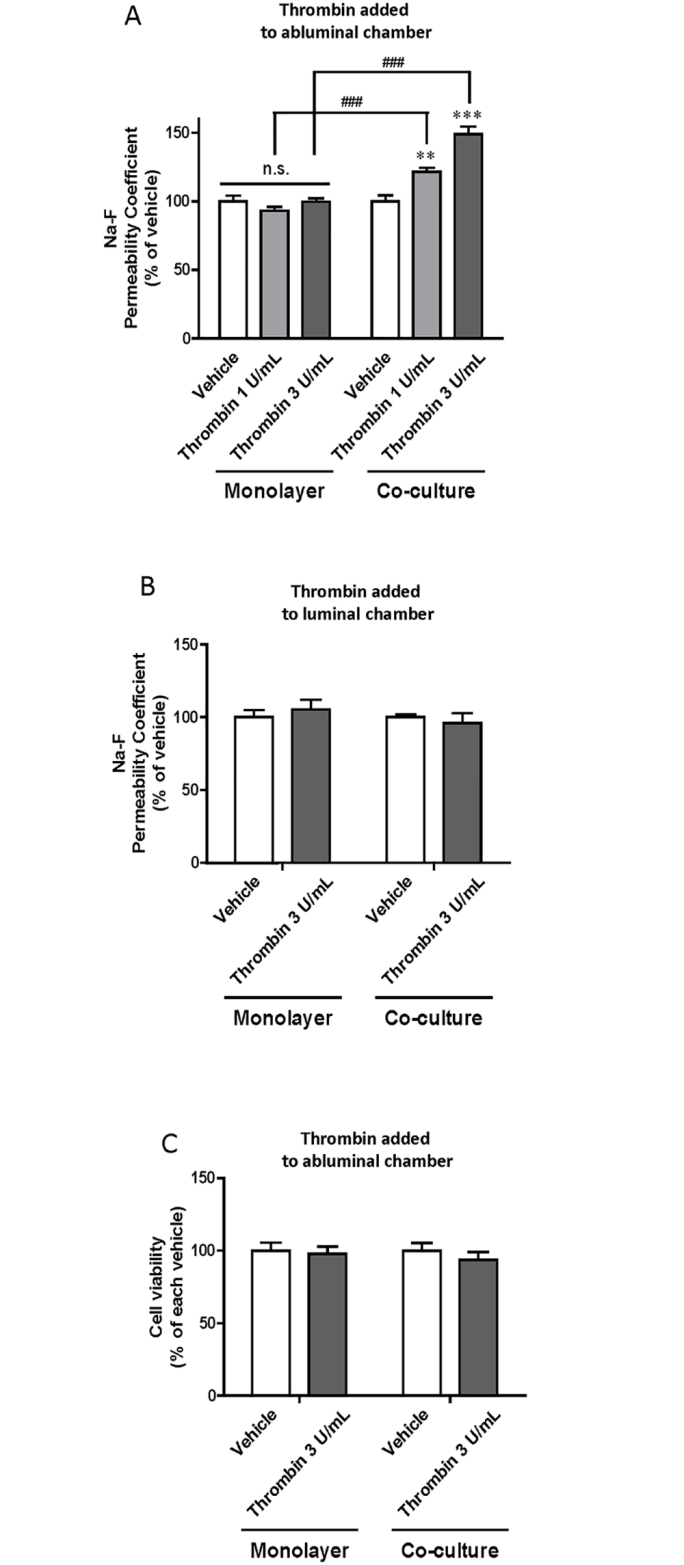
Effects of thrombin on brain endothelial barrier function in the absence or presence of pericytes. Two types of blood–brain barrier models, rat brain microvascular endothelial cell (RBEC) monolayers and RBEC/pericyte co-cultures, were treated by thrombin (1 or 3 U/mL) addition to abluminal (A) or luminal (B) chambers for 24 h. Barrier function was then evaluated by Na-F permeability. (C) Effect of thrombin (3 U/mL) added to abluminal chambers on viability of RBECs in RBEC monolayers and RBEC/pericyte co-cultures. Cell viability of RBECs was assessed using a WST-8 assay. Data are expressed as a percentage of the vehicle control group. *p<0.05, **p<0.01, ***p<0.001 vs. vehicle, ##p<0.01, ###p<0.001 vs each thrombin-treated group (A: n = 14–16, B: n = 8–12, C: n = 7).

RBEC monolayers or RBEC/pericyte co-cultures were treated with thrombin by addition to the abluminal chambers. In the RBEC/pericyte co-cultures, treatment with 1 or 3 U/mL thrombin significantly increased Na-F permeability of the RBECs to 121.59 ± 2.83% and 148.95 ± 5.64% of vehicle-treated co-cultures, respectively, whereas there were no effects of either concentration of thrombin in RBEC monolayers (Fig 4A). TEER values of samples treated with vehicle, 1 U/mL thrombin and 3 U/mL thrombin were 71.4 ± 4.3, 64.4 ± 4.1 and 54.5 ± 1.9 Ω×cm^2^, respectively, in RBEC monolayers. The corresponding values in RBEC/pericyte co-cultures were 66.7 ± 3.6, 54.7 ± 3.6 and 41.1 ± 2.0 Ω×cm^2^, respectively. Then, to examine the effect of thrombin contained in the blood on BBB permeability, we treated RBEC monolayers or RBEC/pericyte co-cultures with thrombin by adding it to the luminal chambers. This had no effect on RBEC Na-F permeability of either RBEC monolayers (105.44 ± 6.66% of vehicle-treated monolayer) or RBEC/pericyte co-cultures (96.00 ± 6.77% of vehicle-treated co-culture) (Fig 4B). Cell viability was not affected by adding thrombin to the abluminal chambers of either RBEC monolayers (97.99 ± 4.77% of vehicle) or RBEC/pericyte co-cultures (93.58 ± 5.57% of vehicle) (Fig 4C).

### Effect of thrombin on the localization and expression level of tight junction proteins

**Fig 5:**
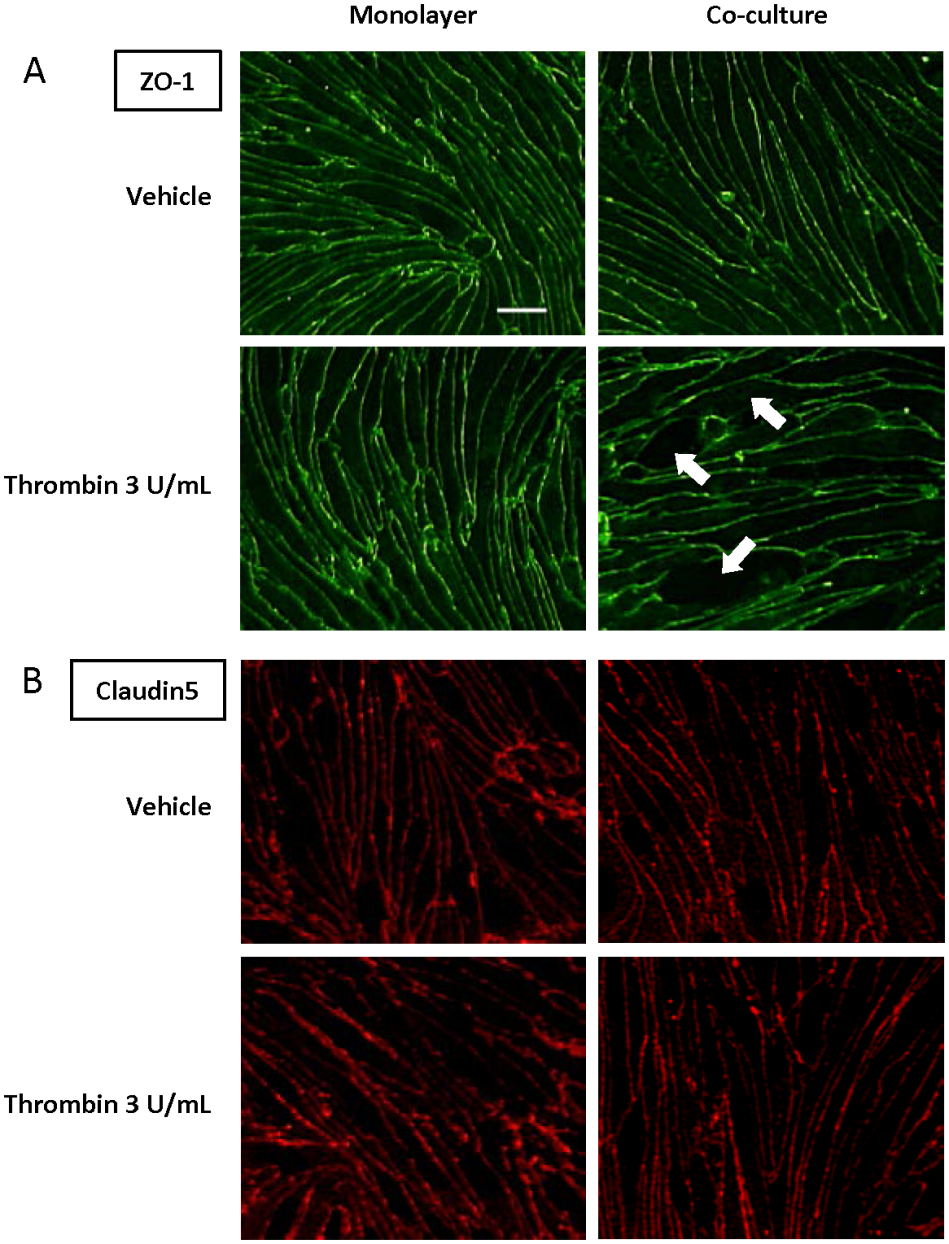
Immunofluorescence staining for tight junction proteins expressed by rat brain microvascular endothelial cells (RBECs) in RBEC monolayers and RBEC/pericyte co-cultures. RBEC monolayers and RBEC/pericyte co-cultures were treated by addition of thrombin (3 U/mL) to abluminal chambers for 24 h. RBECs on trans-well membranes were immunostained (green: ZO-1 (A), red: claudin-5 (B)). Arrows indicate areas of altered localization of ZO-1. Scale bar: 20 μm.

**Fig 6:**
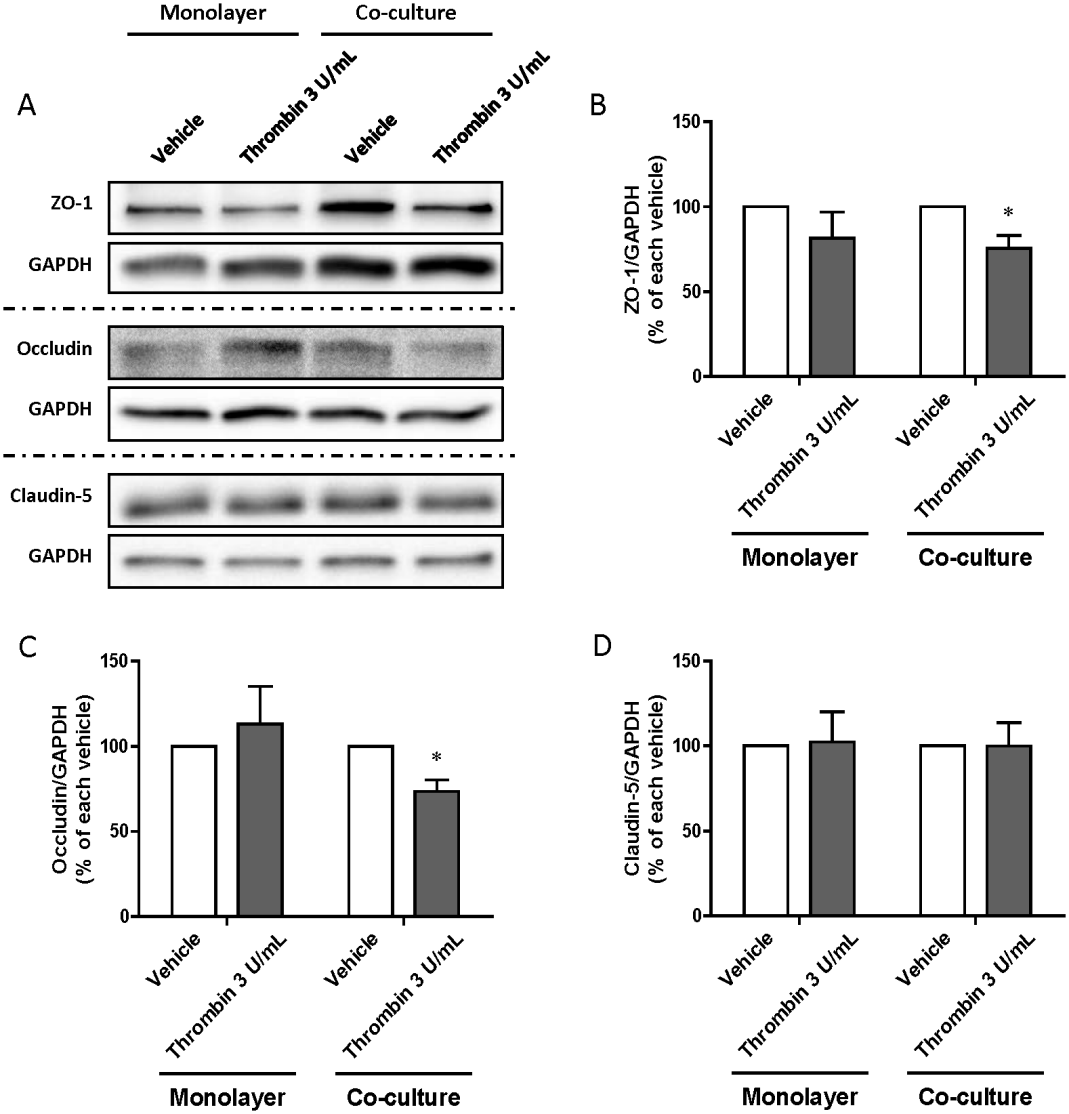
Western blot analysis of tight junction protein expression by rat brain microvascular endothelial cells (RBECs) in RBEC monolayers and RBEC/pericyte co-cultures. RBEC monolayers or RBEC/pericyte co-cultures were treated by addition of thrombin (3 U/mL) to abluminal chambers for 24 h. (A) Representative immunoblots of tight junction proteins (ZO-1, occludin and claudin-5). (B-D) Quantitative analysis of the immunoblots using densitometry. Data are mean ± SEM (n = 3) *p<0.05 vs. each vehicle-treated group.

As shown in Fig 5A and 5B, immunostaining for TJ proteins (zona occludens-1 (ZO-1), claudin-5) revealed a continuous and narrow distribution of these proteins along the cell borders in vehicle-treated RBEC monolayers and RBEC/pericyte co-cultures. This linear distribution of ZO-1 was fragmented and the density of ZO-1 protein expression was reduced in thrombin-treated RBEC/pericyte co-cultures, but not in thrombin-treated RBEC monolayers (Fig 5A), indicating damage to the TJ. These marked changes were not observed in any treatment group after immunostaining for claudin-5 (Fig 5B). In RBEC/pericyte co-cultures, addition of thrombin to the abluminal chamber significantly reduced protein expression of ZO-1 and occludin by 24% and 27%, respectively, but failed to decrease those of claudin-5 (ZO-1; 75.71 ± 7.46% of vehicle, occludin; 73.51 ± 6.76% of vehicle, claudin-5; 99.94 ± 13.78% of vehicle). There were no significant changes in expression of these proteins in thrombin-treated RBEC monolayers (Fig 6A–6D).

### Production of pro-inflammatory cytokines in thrombin-treated brain pericytes

**Fig 7:**
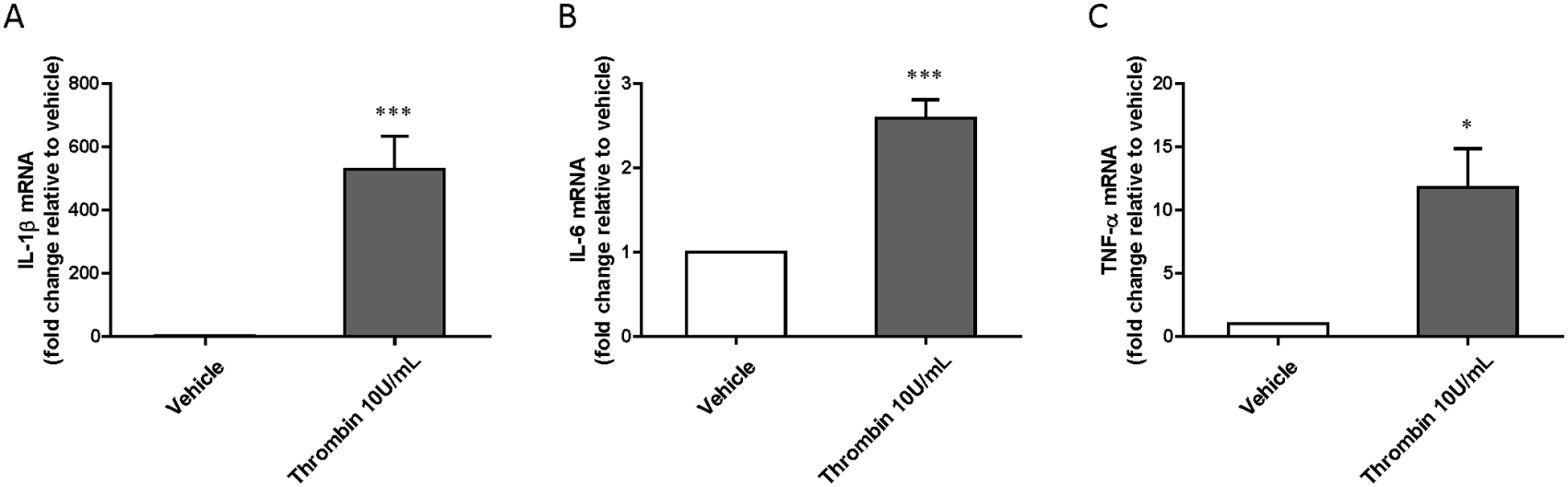
mRNA expression levels of proinflammatory cytokines in brain pericytes exposed to thrombin for 24 h. (A) interleukin-1β (IL-1β), (B) interleukin-6 (IL-6) and (C) tumor necrosis factor-α (TNF-α). Total mRNA of brain pericytes was used for quantitative real-time RT-PCR analysis. Data are mean ± SEM (n = 8). *p<0.05, ***p<0.001 vs vehicle-treated pericytes.

A 24 h-exposure of brain pericytes to thrombin (10 U/mL) significantly up-regulated mRNA expression of IL-1β, IL-6, and TNF-α compared to vehicle-treated pericytes (IL-1β: 529 ± 104, IL-6: 11.8 ± 3.07, TNF-α: 2.59 ± 0.217 -fold increase versus vehicle) (Fig 7A–7C).

## Discussion

The findings of the present study are summarized as follows. (1) Mice fed a HFD for 8W showed both increased weight gain and impaired glucose tolerance. (2) BBB permeability was increased in mice fed a HFD for 8W, accompanied by higher thrombin levels in plasma and brain, compared with in mice fed a ND for 8W. (3) Thrombin penetrated into the brain through the BBB. (4) Thrombin treatment of brain pericytes induced RBEC hyperpermeability. (5) Thrombin treatment of brain pericytes caused fragmented immunostaining for ZO-1 at the intercellular borders of RBECs and decreased ZO-1 and occludin protein levels in RBECs. (6) Thrombin stimulated brain pericytes to express pro-inflammatory cytokines, including IL-1β, IL-6, and TNF-α.

In the present study, we generated a mouse model of obesity by feeding a HFD. This model is considered to be a suitable experimental model for the early phase of pathogenesis of type 2 diabetes induced by obesity as a lifestyle factor. HFD-feeding for 2W and 8W resulted in increased weight gain and impaired glucose tolerance was evident in mice fed a HFD for 8W but not 2W. This result demonstrates that 8W of HFD-feeding cause obesity and insulin resistance in mice.

We evaluated the permeability of the BBB to Na-F in these mice and found that brain uptake of Na-F was significantly increased in 8W but not 2W HFD-fed mice (Fig 1). The K_in_ value of Na-F in mice fed a ND indicated that the BBB in 2W ND-fed mice (7 weeks old) was leaky compared with that in 8W ND-fed mice (13 weeks old), suggesting that the barrier properties of the BBB developed with age during the feeding period. Increased BBB permeability has been previously observed in patients with type 2 diabetes, streptozotocin-induced diabetic model rats and mice fed a HFD for 8W [23, 24, 42], consistent with our present study. Thrombin is known to be increased in the circulation of diabetic patients [31], but levels in the diabetic brain have not yet been determined. Plasma prothrombin/thrombin concentrations were higher in mice fed HFD than with ND, at both 2W and 8W. Levels of brain prothrombin/thrombin in 8W HFD-fed mice were the same as those in 2W ND- and HFD-fed mice, but were lower in 8W ND-fed mice (Fig 2). Uptake of i.v. injected ^125^I-thrombin in normal mice demonstrated that thrombin could cross the BBB from the peripheral blood into the brain, under normal physiological conditions (Fig 3A). Blood-to-brain thrombin transport was increased by excess unlabeled thrombin (Fig 3B), suggesting that an efflux system has considerable activity in thrombin transport at the BBB, although thrombin is accumulated as a result of the net influx of thrombin into the brain under physiological conditions. These findings indicated that the 8W HFD-fed mice experienced long-lasting brain exposure to thrombin, probably because the thrombin efflux system was impaired by HFD feeding. It is, therefore, conceivable that BBB dysfunction develops between 2W and 8W of HFD-feeding and is associated with a long-lasting brain exposure to constant thrombin levels. Although we did not separate brain parenchymal from vascular uptake of ^125^I-thrombin, co-injection with an excess amount of unlabeled thrombin, to prevent ^125^I-thrombin binding to brain endothelium, increased brain uptake of ^125^I-thrombin. This suggested that increased binding of ^125^I-thrombin to brain endothelium did not occur in parallel with its increased brain parenchymal uptake. Further experiments, using brain capillary depletion analysis, are necessary to measure accumulation of ^125^I-thrombin in brain endothelium.

The possibility that thrombin was synthesized in the brain in our experiments could not be excluded. There are some reports showing that brain microvessels and brain endothelial cells are a source of thrombin in the brain. Grammas *et al* reported that brain endothelial cells, when treated with an inflammatory protein cocktail containing IL-1β, IL-6, TNF-α, interferon-γ and lipopolysaccharide, released thrombin into the culture medium [43]. This finding suggests that brain endothelial cells or brain microvessels are a potential source of increased brain thrombin during diabetes, given that diabetes is associated with increased circulating levels of various inflammatory proteins mentioned above [3, 44]. Furthermore, our previous study suggested that brain pericytes located adjacent to these cells are important in the response to thrombin and thus BBB dysfunction [36].

To elucidate whether thrombin in the brain inhibits BBB function and whether brain pericytes are involved in this process, we used two types of *in vitro* BBB model: RBEC monolayers and RBEC/pericyte co-cultures. As shown in Fig 4, thrombin added to the abluminal chambers increased RBEC permeability to Na-F only in RBEC/pericyte co-cultures, but not in RBEC monolayers, without affecting cell viability. In contrast, thrombin added to the luminal chambers failed to change Na-F permeability either in RBEC monolayers or RBEC/pericyte co-cultures. Although a higher dose of thrombin (60 U/mL) was previously shown to directly impair the brain endothelial barrier [35], our results indicate that brain pericytes respond to thrombin by reducing endothelial barrier integrity. We next examined whether brain pericytes treated with thrombin demonstrate altered expression of TJ proteins in RBECs. In the presence of thrombin-treated pericytes, the linear distribution of ZO-1-like immunoreactivity along the intercellular borders of RBECs became fragmented and decreased protein levels of ZO-1 and occludin were observed (Fig 5). These results are also consistent with a study of experimental diabetic rodent models [21, 45]. MMP-9 contributes to BBB dysfunction by degrading TJ proteins under pathological conditions such as ischemic stroke [45, 46] and we have reported that brain pericytes, in particular among the cell types that constitute the BBB, release MMP-9 after thrombin stimulation [36]. MMP-9 is therefore a strong candidate to be a mediator of the thrombin-induced effect on BBB dysfunction.

As shown in Fig 7, mRNA levels of IL-1β, IL-6, and TNF-α in brain pericytes were significantly upregulated by thrombin treatment. These pro-inflammatory cytokines have been shown to increase brain endothelial permeability *in vitro* and *in vivo* [47–50] and may represent a second mechanism whereby thrombin-treated brain pericytes may be responsible for increased BBB permeability. However, the involvement of these pro-inflammatory cytokines in BBB dysfunction during diabetes must be confirmed by additional studies. Nevertheless, considering present and previous findings together, thrombin-reactive brain pericytes are likely to make an important contribution to the development of BBB dysfunction associated with vulnerable endothelial TJs in diabetes.

In the present study, we have shown that HFD-induced prediabetic conditions were associated with BBB hyperpermeability, caused by elevated plasma thrombin levels that led to chronic brain exposure to constant levels of thrombin and that thrombin is able to be transported from circulating blood into brain across the BBB and also produced in the brain. Thrombin here could represent a possible biomarker to detect impaired integrity of the BBB prior to the onset of neurological disorders. In addition, we have shown that thrombin in the brain stimulates brain pericytes to induce BBB dysfunction. These findings imply that a thrombin-pericyte interaction in the brain could be a significant mechanism responsible for BBB dysfunction under obesity-associated diabetic conditions and thus could represent a therapeutic target for CNS complications of diabetes.
